# 

**Published:** 1820-01

**Authors:** 


### MONTHLY CATALOGUE OF MEDICAL BOOKS.

Sound Mind; or, Contributions to the Natural History and Physiology of the Human Intellect. By John Haslam, M.D. 8vo. 7s.

A Sketch of the History and Cure of Contagious Fever. By Robert Jackson, M.D. 8vo. 8s.

A Sketch of the Economy of Man. 8vo. 10s. 6d.

A Compendium of Anatomy, Human and Comparative. By Andrew Fyfe. 4 vols. with Plates. The seventh Edition. 8vo. 2l. 2s.

Elements of Physiology. By A. B. Richeraud. Translated from the French, by G. J. M. De Lys, M.D. A new Edition. 8vo. boards, 12s.

A Treatise on Nervous Diseases. By John Cooke, M.D. F.A.S. &c. Vol. I. on Apoplexy, including Apoplexia Hydrocephalica, or Water in the Head. 8vo. 12s. boards.

The Medical Intelligencer, or Monthly Analytical Compendium. No. II. 8d.

